# How leaders' bias tendency affects employees' knowledge hiding behavior: The mediating role of workplace marginalization perception

**DOI:** 10.3389/fpsyg.2022.965972

**Published:** 2022-08-05

**Authors:** Sijin Du, Wenli Xie, Jianjun Wang

**Affiliations:** ^1^School of Finance and Economics, Qinghai University, Xining, China; ^2^School of Marxism, Heilongjiang University, Harbin, China; ^3^School of Finance and Economics, Qinghai University, Xining, China

**Keywords:** leaders' bias tendency, employees' knowledge hiding behavior, workplace marginalization perception, leader-member exchange theory, resource conservation theory

## Abstract

Employees' knowledge hiding behavior has an essential inhibitory impact on organizational innovation and employee knowledge sharing. Accordingly, studying the antecedents and influencing mechanisms of employees' knowledge hiding behavior is quite necessary. In the perspective of leader–member exchange theory and resource conservation theory, the leaders' bias tendency will lead to the workplace marginalization perception of some employees and promote the generation of employees' knowledge hiding behavior. Thus, this research is intended to discuss the influence of leaders' bias tendency toward employees' knowledge hiding behavior, and to analyze the mediating effects of employees' perception of workplace marginalization and the moderating role of emotional commitment to the organization. The sample of this study covered 500 Chinese full-time corporate employees. The conclusions of the research indicate that the following: (1) Leaders' bias tendency is vitally and absolutely correlated with employees' knowledge hiding behavior; (2) Workplace marginalization perception plays an intermediary role between leaders' bias tendency and employees' knowledge hiding behavior; (3) Emotional commitment to the organization plays a negative moderating role between leaders' bias tendency and employees' knowledge hiding behavior; (4) Emotional commitment to the organization plays a negative moderating role between workplace marginalization perception and employees' knowledge hiding behavior. These findings will help organizations and managers to recognize the harm of bias tendency, regulate their own behaviors, and effectively reduce the generation of employees' knowledge hiding behaviors, thereby promoting knowledge sharing and innovative behaviors in organizations.

## Introduction

Employees' knowledge hiding behavior is defined as the behavior of individuals who intentionally conceal learning in the organization confronts knowledge inquiries from others (Connelly et al., 2012). A study by Riege (2005) showed that knowledge sharing and knowledge communication among employees can effectively promote the improvement of management efficiency, thereby cutting the management cost of the organization and increasing the innovation efficiency of the organization. However, not all labors would like to share knowledge in the specific workplace. For example, a survey of Peng (2012) shows that 46% of employees in China had experienced employees' knowledge hiding behavior. In addition, a study by AMR also showed that although corporations are ready to pay $73 billion per year on knowledge management and learning dissemination, there are even people who are unwilling to share knowledge (Martine, 2010). This shows that employees' knowledge hiding behavior is universal. Thus, it is particularly material to comprehend the causes that generate employees' knowledge hiding behavior. Numerous studies have explored the reasons of employees' knowledge hiding behaviors, such as interpersonal suspicion (Connelly et al., 2012; Jiang and He, 2013), psychological ownership of knowledge (Peng, 2013), employees' open personality (Teh et al., 2011; Lin and Wang, 2012; Anand and Jain, 2014), perception of organizational competitive climate (Yang and Tang, 2018), and sense of procedural fairness (Zhou et al., 2020).

However, the influence of organizational leadership style on employees' knowledge hiding behavior has not been fully discussed (Tian et al., 2021). Whether in China or in other countries, leaders' bias tendency is a leadership style and a common phenomenon in the workplace (Liden and Maslyn, 1998; Zhou et al., 2020). Leaders' bias tendency is defined as the standard of specific “individual-oriented leadership behavior” demonstrated by leaders confronted distinct subordinates (Wu et al., 2010). A study by Gao and Wang (2018) showed that the leaders' bias tendency are culturally adaptive and universal in China. However, the influence mechanism of the leaders' bias tendency on employees' knowledge hiding behavior is still unclear. Therefore, it is particularly important for this study to investigate the mechanisms of the leaders' bias tendency toward employees' knowledge hiding behavior.

On the basis of the leader–member exchange theory and resource conservation theory, this study argues that workplace marginalization perception has a prominent mediating effect on the leaders' bias tendency on employees' knowledge hiding behavior. As the “outsiders” of the informal collective of leaders, these employees cannot be treated fairly by the leaders in the distribution of opportunities and resources, and are more likely to be excluded by the “insiders.” Therefore, employees are more likely to feel workplace marginalization perception in the atmosphere of their leaders' bias tendency. On the other side, it is also complicated for the workers who are marginalized in the workplace to generate motivation to participate in knowledge sharing and organizational innovation in the institution. In the view of resource conservation theory, people's sharing behavior is based on the principle of reciprocity, and resources are invested to maintain the existing resources and generate new resources. Obviously, in the context of the leaders' bias tendency and the workplace marginalization perception, he benefits of the employees' knowledge hiding behavior is clearly greater.

In addition, the influence of specific cultural and social contexts on research should be noted (Mao et al., 2017). In China, the traditional Confucian thought has deeply influenced the character, values, behaviors as well as the norms of Chinese people and has derived Confucian workplace values (Yan et al., 2007). Employees with high level of Confucian workplace values will try their best to obey the organization and authority, and become more loyal (Hwang, 2000), resulting in generating an emotional commitment to the organization. Therefore, we regard emotional commitment to the organization as a specific cultural factor to study the moderating effect of emotional commitment to the organization on employees' knowledge hiding behavior.

Employees with high level of emotional commitment to the organization tend to be more motivated to do things that contribute to the organization's development. Therefore, we believe that the employees with high level of emotional commitment to the organization will make less employees' knowledge hiding behaviors than employees with low level of emotional commitment to the organization under the differential pattern of leaders' bias tendency. On the other hand, under the perception of workplace marginalization perception, employees with high level of emotional commitment to the organization also engage in less employees' knowledge hiding behaviors than the employees with low level of emotional commitment to the organization. The reason is that the employees with high level of emotional commitment to the organization devote more energy to their work, while their low level of emotional commitment to the organization merely includes the contractual connection between the employee and the organization. Therefore, the employees with high level of emotional commitment to the organization will certainly contribute more when it comes to organizational interests.

**Figure 1:**
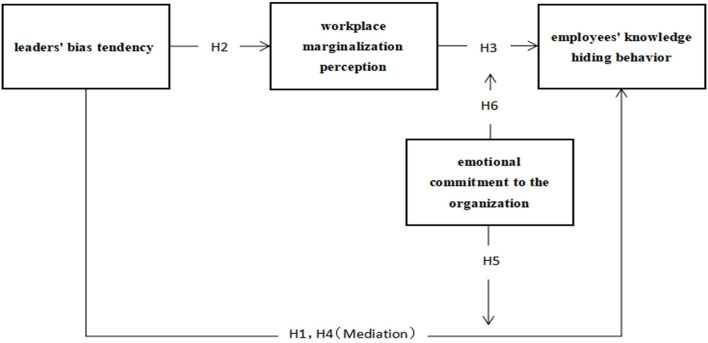
Research model diagram.

The innovations of this manuscript are as follows: (1) In terms of research perspective, this research elucidates the influence of the leaders' bias tendency toward employees' knowledge hiding behavior from the perspective of leadership style and organizational culture. (2) In terms of research methods, this study uses a questionnaire survey with a 4-month interval to collect data, which effectively reduces the error effect of the homologous method. The model is displayed in Figure 1.

## Theory and hypotheses

### Leader–member exchange theory and resource conservation theory

Leader–member exchange theory, clarified as “relationship-based social exchange between leaders and members” (Graen, 1995) is useful for explaining the behavior and psychological states of leaders or employees in the workplace (Kim et al., 2009; Portoghese et al., 2015). This theory believes that with limited resources within organizations, leaders will develop a limited number of subordinates who meet their role expectations or appreciation, giving them more care and trust; thus, forming informal leader-centered groups (Dienesch and Liden, 1986; Loi et al., 2009). Cleyman et al. (2013) condensed the core ideas of leader–member exchange theory. First, due to the constraints of time and resources, leaders cannot have the same relationship with every subordinate, but will selectively establish different relationships with their subordinates according to whether their performance meets the leadership expectations and their own employment preferences (Graen et al., 1986; Liden and Maslyn, 1998). In addition, according to the connection between supervisors and workers, subordinates are divided into “insiders” and “outsiders.” The “insiders” can receive more trust, support, resources, etc., and are more likely to be respected by the leader whereas the “outsiders” may be marginalized in the workplace or gain the perception of workplace marginalization (Deluga and Perry, 1994; Sparrowe and Liden, 1997).

Resource conservation theory was originally proposed as a stress theory by Hobfoll (1989) and applied in the study of psychology and organizational behavior (Hobfoll, 1989). This theory holds that people have a tendency to keep, protect, and obtain resources, and thus, both the latent threat of resource loss and the actual resource loss can generate individual tension and stress (Hobfoll et al., 2018). Hobfoll summarizes several principles of resource conservation theory. First, the impact of losses is greater. For individuals, the influence of resource loss is much more principal than resource acquisition, and its influence is faster and continues longer (Halbesleben and Bowler, 2007; Halbesleben, 2010; Halbesleben and Wheeler, 2011). Second, in the face of the desperate situation of resource exhaustion, individuals' self-protective defense mechanisms will be triggered and they will display aggressive or irrational behavior (Halbesleben et al., 2014). Finally, neither individual nor organization can own resources alone, but influence each other. Therefore, the organizational climate and cultural setting play an indispensable role in resource maintenance (Hobfoll, 2012).

### The influence of the leader's bias tendency on employees' knowledge hiding behavior

Studies have shown that the leaders' bias tendency is widespread in East Asian cultures (Xu et al., 2002). The leaders' bias tendency will occur in the workplace when “employees seek personal friendship with their bosses, and their bosses also actively develop personal connections with themselves” (Sun and Wang, 2013; Gao and Wang, 2018). The leaders' bias tendency is described as whether the leaders are inclined to make corresponding decisions for the purpose of maintaining personal interests or self-interest (Liu et al., 2015). The studies have shown that the leaders' bias tendency will have a negative influence on employees' opinions and manners, such as moral disengagement (Tang et al., 2018), which is not conducive to the realization of group performance (Wu et al., 2010; Jiang et al., 2015), reduce trust in leaders (Kunze et al., 2016), lead to mutual competition, jealousy, relationship conflicts among subordinates (Zhang et al., 2015), and generate subordinates' negative attitude toward leadership and organization (Li et al., 2017).

Because of the leader–member exchange theory, we believe that the leaders' bias tendency will promote the employees' knowledge hiding behavior. The attitudes and behaviors of leaders at work are important factors affecting employees' knowledge hiding behavior (Tang et al., 2015). Employees' knowledge hiding behaviors are associated with inappropriate leadership styles and leadership behaviors. The leaders' bias tendency excludes some employees from leading informal groups and weakens their support, resources, and opportunities (Chen et al., 2014; Yu and Zhang, 2016). If employees are affected by this leadership style and leadership behavior, they will have a negative impact and stop investing their own resources at work, such as weakening employees' willingness to share knowledge (Cai et al., 2013). Based on the above, this research proposes Hypothesis H1 as follows:

H1: There is a positive correlation between the leaders' bias tendency and employees' knowledge hiding behavior.

### The mediating role of workplace marginalization perception

On the basis of the leader–member exchange theory and the resource conservation theory, we believe that the leaders' bias tendency will promote employees' workplace marginalization perception, which will lead to employees' knowledge hiding behavior. The workplace marginalization perception derives from the isolation and exclusion of others in the organization or team (Williams, 1997; Liu et al., 2012), which is one of the consequences of leaders dividing subordinates into “insiders” and “outsiders.” The leader–member exchange theory well explains this phenomenon. The existing research shows that “insiders” enjoy more opportunities, resources and respect, which will make the employees in the circle reluctant to contact employees outside the circle (Odle, 2014), employees outside the circle are more likely to feel aggrieved (Cleyman et al., 2013), team conflict (Boies and Howell, 2006), and colleagues' distrust (Kim et al., 2010). This means that “outsiders” are more likely to feel excluded and isolated, that is, to gain the perception of workplace marginalization.

On the other hand, based on the resource conservation theory, when individuals confront resource loss, they will automatically generate defense mechanisms and show some negative behaviors (DeVente et al., 2003; Melamed et al., 2006). Being perceived as an “outsider” by leaders means that they will enjoy less support, opportunities, and resources in the future, which is typical of individual resource loss (Hobfoll and Shirom, 2001). Therefore, in the face of knowledge consultation and knowledge sharing requests from leaders and even colleagues, employees' knowledge hiding behaviors will occur, thereby preventing further loss of resources and triggering defense mechanisms. Based on the above, the research rises the following hypotheses:

H2: There is a positive correlation between the leaders' bias tendency and the workplace marginalization perception.
H3: There is a positive correlation between the workplace marginalization perception and the employees' knowledge hiding behavior.
H4: Workplace marginalization perception plays a mediating role between leaders' bias tendency and employees' knowledge hiding behaviors.

### The moderating role of emotional commitment to the organization

Leaders' bias tendency and workplace marginalization perception are not a good workplace experience for employees (Williams et al., 2005). However, peoples' perceptions and reactions to leaders' bias tendency and workplace marginalization perception may be affected by employees' emotional commitment to the organization (Tsachouridi and Nikandrou, 2019). This study argues that, for employees, the emotional commitment to the organization will play a moderating role in their leaders' bias tendency on the employees' knowledge hiding behaviors. Similarly, emotional commitment to the organization will also play a moderating role in the workplace marginalization perception on employees' knowledge hiding behavior.

Employees' emotional commitment to the organization is mainly affected by factors such as organizational goals, organizational climate, and cultural values (Meyer and Allen, 1991). Emotional commitment to the organization represents an employee's emotional attachment to the organization and the state of work engagement (Blomme et al., 2012). In the differential atmosphere of the leaders' bias tendency, employees who are excluded from the informal group of the leader will have a great sense of gap and workplace marginalization perception. This is distressing and unacceptable for employees with low level of emotional commitment to the organization (Hobfoll and Shirom, 2001) because they have insufficient emotional attachment and loyalty to the organization itself. Therefore, it is difficult for employees with low level of emotional commitment to the organization to consider the interests of the organization and the development of the organization, and a certain defense mechanism will be developed against the leaders' bias tendency, forming negative behaviors such as employees' knowledge hiding behavior. Moreover, individuals with a high level of emotional commitment to the organization have a strong sense of attachment and loyalty to the organization, and they are more able to overcome the perceived pain of leaders' bias tendency and workplace marginalization perception so as to do things that are beneficial to the organization (Eisenberger et al., 1990; Riketta and Landerer, 2005). Therefore, individuals with a high level of emotional commitment to the organization will weaken the influence of leaders' bias tendency and the workplace marginalization perception on employees' knowledge hiding behavior. On the basis of the above, this study proposes the following hypotheses:

H5: Emotional commitment to the organization plays a negative moderating role between leaders' bias tendency and employees' knowledge hiding behaviors.
H6: Emotional commitment to the organization plays a negative moderating role between workplace marginalization perception and employees' knowledge hiding behavior.

## Materials and methods

### Participants and procedures

This research was conducted in a number of companies in Jiangsu Province, with help and support from managers. An entire of 542 company employees joined in the research. They received this questionnaire at their workstation during working hours, and one of the authors participated in the recovery of the questionnaire. To obtain the support of the surveyed staff, the author made a pledge to all participants that the questionnaire was anonymous and would be used for academic purposes only and not for other purposes. Also, this clause was written into the preface of the questionnaire.

So as to abbreviate the influence of the homologous approach on this study, the study was conducted in two separate studies with a 4-month interval. In the first survey, 542 participants were required to supply demographic details such as age, gender, and their level of education, in addition to assessing their leaders' bias tendency and emotional commitment to the organization. The second survey, conducted 4 months after the first, focused on collecting information about participants' workplace marginalization perception and employees' knowledge hiding behaviors in the over 4 months. In the end, the author recovered 500 effective questionnaires, with a practical rate of 92.25%.

In the survey sample, there are 244 males (48.8%) and 256 females (51.2%) participated, whose ages are mainly in the age group 25–30-years and 30–40-years (33.2 and 28.6%, respectively); their education level is mainly undergraduate and associate, and master's degree level (42.6 and 24.2%, respectively).

### Measures

The questionnaires in this study were compiled from mature scales that have been validated and used many times to guarantee the authority and validity of the scales. Since most of the respondents are Chinese employees, to help them understand the English scale, the research team invited two English-major graduate students to translate the scale, and one of the authors personally proofread it. Except for the control variable, all variable scales were scored using a 5-point Likert scale, with 1 indicating “strongly disagree” and 5 indicating “strongly agree.”

#### Leaders' bias tendency

Used the leaders' bias tendency scale developed by Liu et al. (2015); the scale comprised a total of five questions, such as “For the promotion of subordinates, I usually only consider those subordinates that I appreciate,” and so on.

#### Workplace marginalization perception

Used the workplace marginalization perception scale developed by Liu et al. (2012); the scale comprised four questions, such as “leaders usually don't take my advice,” and so on.

#### Employees' knowledge hiding behavior

Used the employees' knowledge hiding behavior scale developed by Connelly et al. (2012); the scale has a total of 12 questions, such as “I may answer that I don't know, although I know,” and so on.

#### Emotional commitment to the organization

Using the organizational affective commitment scale developed by Yao et al. (2008), the scale has four questions such as “I think I have a sense of belonging in this company,” and so on.

#### Control variable

In the study, control variables included gender, age, and education level. Existing studies have demonstrated that these variables may have an impact on the dependent variables of this study.

## Results

### Reliability analysis

We tested the reliability of the scale using SPSS v.22.0 with reference to the method of Eisinga et al. (2013). The outcomes of the reliability analysis are shown in Table 1. The Cronbach's-α coefficients of the four variables of leaders' bias tendency, workplace marginalization perception, employees' knowledge hiding behavior, and emotional commitment to the organization are all greater than 0.8. The combined reliability of AVE and CR of the four variables, AVE are all higher than 0.8. Greater than 0.5, the CR is greater than 0.7, which ensures the reliability of the reliability.

**Table 1:** Reliability analysis.

**Variables**	**Cronbach's-α coefficient**	**AVE**	**CR**
Leaders' bias tendency	0.859	0.552	0.860
Workplace marginalization perception	0.803	0.506	0.803
Employees' knowledge hiding behavior	0.937	0.559	0.938
Emotional commitment to the organization	0.827	0.549	0.829

### Confirmatory factor analyses

We performed confirmatory factor analysis using AMOS 24.0 to determine the discriminant validity between variables and the reasonableness of the setting. According to a related research by Hu and Bentler (1999), the model fits well when χ^2^/*df* < 3, GFI > 0.90, RMR < 0.05, NFI > 0.90, CFI > 0.90, TLI > 0.90, and RMSEA < 0.08. The confirmatory factor analysis of the four variables of leaders' bias tendency, workplace marginalization perception, employees' knowledge hiding behavior, and emotional commitment to the organization are shown in Table 2. The fit index of the 4-factor model (χ^2^/*df* = 1.180, CFI = 0.992, NFI = 0.951, GFI = 0.952, TLI = 0.991, RMR = 0.048, and RMSEA = 0.019) was better than other factor models, implying that these four variables have significant discriminant validity.

**Table 2:** Confirmatory factor analyses.

	**χ^2^**	***df***	**χ^2^/*df***	**CFI**	**NFI**	**GFI**	**TLI**	**RMR**	**RMSEA**
Four-factor model^g^	317.354	269	1.180	0.992	0.951	0.952	0.991	0.048	0.019
Three-factor model^f^	1069.875	272	3.933	0.871	0.835	0.817	0.857	0.144	0.077
Three-factor model^e^	906.016	272	3.331	0.897	0.860	0.849	0.887	0.108	0.068
Three-factor model^d^	711.345	272	2.615	0.929	0.890	0.877	0.922	0.098	0.057
Two-factor model^c^	1297.304	274	4.735	0.834	0.800	0.785	0.819	0.137	0.087
Two-factor model^b^	1467.418	274	5.356	0.807	0.773	0.756	0.788	0.182	0.093
One-factor model^a^	2134.010	275	7.760	0.699	0.670	0.664	0.672	0.185	0.116

a Leaders' bias tendency + workplace marginalization perception + employees' knowledge hiding behavior + emotional commitment to the organization.

b Leaders' bias tendency + emotional commitment to the organization; workplace marginalization perception + employees' knowledge hiding behavior.

c Leaders' bias tendency; emotional commitment to the organization + workplace marginalization perception + employees' knowledge hiding behavior.

d Leaders' bias tendency; emotional commitment to the organization; workplace marginalization perception + employees' knowledge hiding behavior.

e Leaders' bias tendency; workplace marginalization perception; emotional commitment to the organization + employees' knowledge hiding behavior.

f Leaders' bias tendency; workplace marginalization perception + emotional commitment to the organization; employees' knowledge hiding behavior.

g Leaders' bias tendency; workplace marginalization perception; employees' knowledge hiding behavior; emotional commitment to the organization.

### Common method deviation test

To prevent the influence of homologous methods on this study, the researchers used the method of sectional investigation every 4 months to eliminate this influence. In addition, to exclude the influence of homologous methods, we chose to use Harmer's univariate test to decide whether there is an ordinary means prejudice, according to a related study by Ding and He (2004). The test results indicated that the first principal component of the non-rotating Harmer single factor test was 32.129% (less than 40%), so the homologous method deviation could be excluded.

### Results of descriptive statistical analysis

In the descriptive statistics and correlation analysis section, we first calculated the MEAN and SD of each variable. Next, we calculated the control variables and the Pearson correlation between the variables according to the method of Hauke and Kossowski (2011). The descriptive statistics of this study are shown in Table 3.

**Table 3:** Results of descriptive statistical analysis.

**Variables**	**MEAN**	**SD**	**1**	**2**	**3**	**4**	**5**	**6**	**7**
1. Gender	1.512	0.500	1						
2. Age	2.646	1.241	−0.040	1					
3. Education level	3.300	1.101	−0.036	−0.481^***^	1				
4. Leaders' bias tendency	3.095	1.028	0.011	0.019	0.012	1			
5. Workplace marginalization perception	2.931	0.987	−0.000	0.030	0.018	0.400^***^	1		
6. Employees' knowledge hiding behavior	2.735	0.929	−0.023	−0.009	0.003	0.342^***^	0.521^***^	1	
7. Emotional commitment to the organization	2.788	0.997	0.024	−0.036	−0.023	−0.055	−0.062	−0.359^***^	1

^*^p < 0.05, ^**^p < 0.01, and

*** p < 0.001.

The research results show that the leaders' bias tendency is positively correlated with the workplace marginalization perception, and the correlation coefficient is 0.400 (*p* < 0.001); the leaders' bias tendency is positively correlated with employees' knowledge hiding behavior, and the correlation coefficient is 0.342 (*p* < 0.001); workplace marginalization perception is positively correlated with employees' knowledge hiding behavior, and the correlation coefficient is 0.521 (*p* < 0.001). These results provide the support for the following data analysis work.

### Hypothesis testing

When testing the mediating effect and moderating effect, we adopted the test methods of Preacher and Hayes (2004); Hayes (2013), and used the Process plugin of SPSS to examine the mediating effect and moderating effect. We carried out multiple regression analysis and process analysis in the hypothetical model to test the direct and indirect influence of leaders' bias tendency on employees' knowledge hiding behavior. The results are shown in Table 4. There is a positive correlation between the leaders' bias tendency and employees' knowledge hiding behavior (*β* = 0.310, *p* < 0.001), supporting Hypothesis H1; there is an influential positive correlation between leaders' bias tendency and workplace marginalization perception (*β* = 0.383, *p* < 0.001), supporting Hypothesis H2; there is an essential positive correlation between workplace marginalization perception and employees' knowledge hiding behavior (*β* = 0.432, *p* < 0.001), supporting Hypothesis H3.

**Table 4:** Hypothesis testing.

**Variables**	**Dependent variable: workplace**		**Dependent variable: employees'**
	**marginalization perception**		**knowledge hiding behavior**
	**Model 1**	**Model 2**	**Model 3**
**Control variable**			
Gender	−0.004	−0.051	−0.050
Age	0.029	−0.017	−0.030
Education level	0.028	−0.011	−0.023
**Independent variable**			
Leaders' bias tendency	0.383^***^	0.310^***^	0.145^***^
**Mediating variable**			
Workplace marginalization perception			0.432^***^
*R*	0.161	0.118	0.295
Δ*R*	0.155	0.111	0.287
*F*	*F*_(4, 495)_ = 23.823^***^	*F*_(4, 495)_ = 16.604^***^	*F*_(5, 494)_ = 41.259^***^

^*^p < 0.05, ^**^p < 0.01, and

*** p < 0.001.

Finally, the process results show that employees' ingratiating conduct in the workplace has an indirect impact on emotional exhaustion through work stress [*β* = 0.166, 95% confidence interval (0.141, 0.227), excluding 0]. Therefore, it is assumed that H4 is supported.

### Moderating effect test

The test results of the moderating effect are shown in Table 5. (1) In Model 3, the regression coefficient of the leaders' bias tendency × emotional commitment to the organization and employees' knowledge hiding behavior is −0.329 (*p* < 0.001), reaching a significant level, which indicates that the emotional commitment to the organization plays a negative moderating role between the leaders' bias tendency and employees' knowledge hiding behavior. Hypothesis H5 is supported; (2) In model 6, the regression coefficient of workplace marginalization perception × emotional commitment to the organization and employees' knowledge hiding behavior is −0.299 (*p* < 0.001), reaching a significant level, which indicates that emotional commitment to the organization plays a negative moderating role between workplace marginalization perception and employees' knowledge hiding behavior. Hypothesis H6 is supported.

**Table 5:** Moderating effect testing.

**Variables**	**Dependent variable: employees' knowledge hiding behavior**					
	**Model 1**	**Model 2**	**Model 3**	**Model 4**	**Model 5**	**Model 6**
**Control variable**						
Gender	−0.051	−0.038	−0.032	−0.046	−0.034	−0.044
Age	−0.017	−0.032	−0.026	−0.029	−0.043	−0.032
Education level	−0.011	−0.025	−0.040	−0.021	−0.035	−0.034
**Independent variable**						
Leaders' bias tendency	0.310^***^	0.294^***^	0.283^***^			
**Mediating variable**						
Workplace marginalization perception				0.492^***^	0.474^***^	0.446^***^
**Moderating variable**						
Emotional commitment to the organization		−0.319^***^	−0.372^***^		−0.308^***^	−0.329^***^
**Interactive term**						
Leaders' bias tendency × Emotional commitment			−0.329^***^			
to the organization						
Workplace marginalization perception ×						−0.299^***^
**Emotional commitment to the organization**						
*R* ^2^	0.118	0.235	0.360	0.273	0.381	0.478
Δ*R*^2^	0.118	0.117	0.125	0.273	0.108	0.096
*F*	16.604^***^	30.336^***^	46.216^***^	46.479^***^	60.874^***^	75.157^***^
Δ*F*	16.604^***^	75.293^***^	96.341^***^	46.479^***^	86.384^***^	91.076^***^

^*^p < 0.05, ^**^p < 0.01, and

*** p < 0.001.

### Simple efficiency analysis and simple efficiency diagram

**Figure 2:**
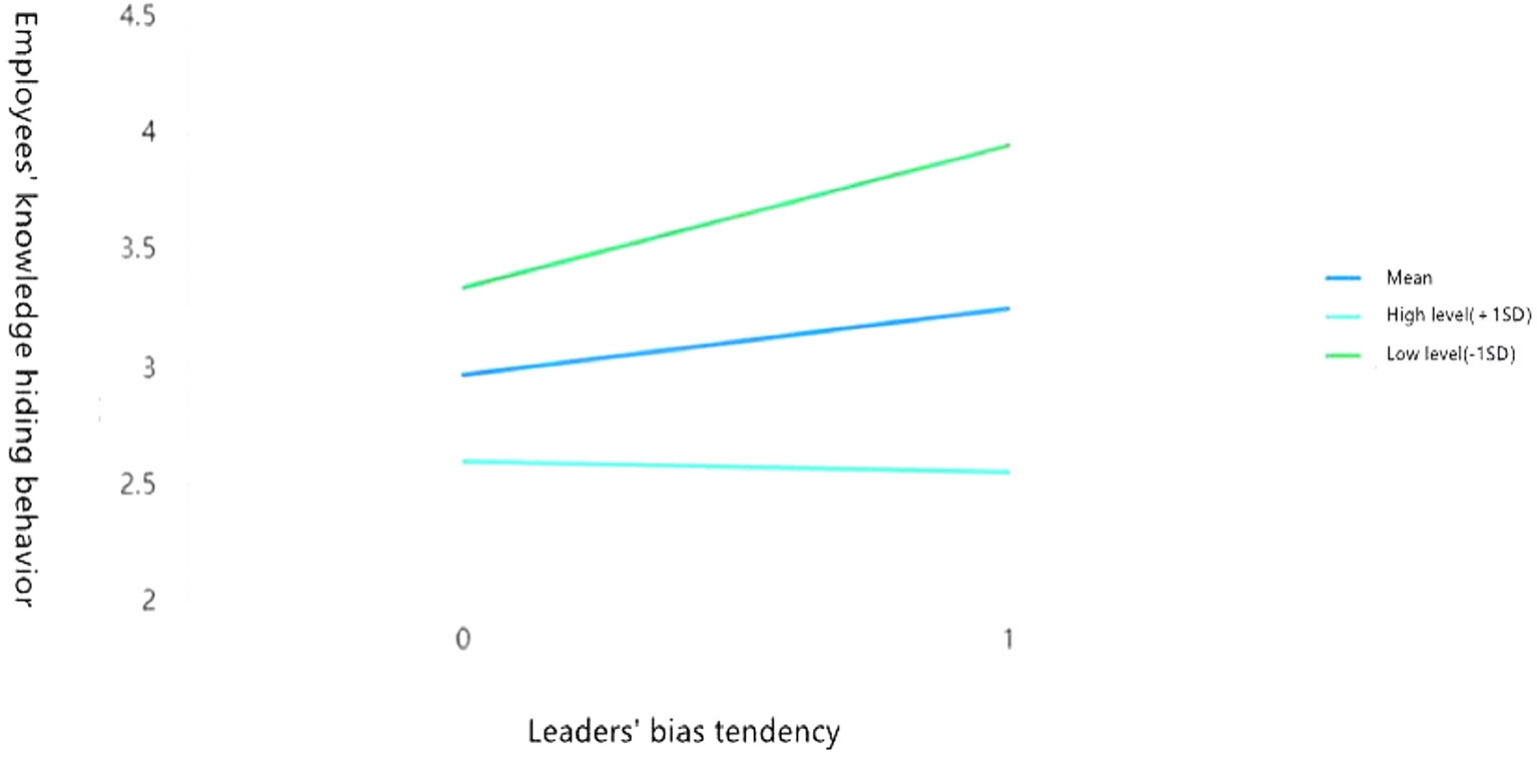
Emotional commitment to the organization plays a negative moderating role between the leaders' bias tendency and employees' knowledge hiding behavior.

**Figure 3:**
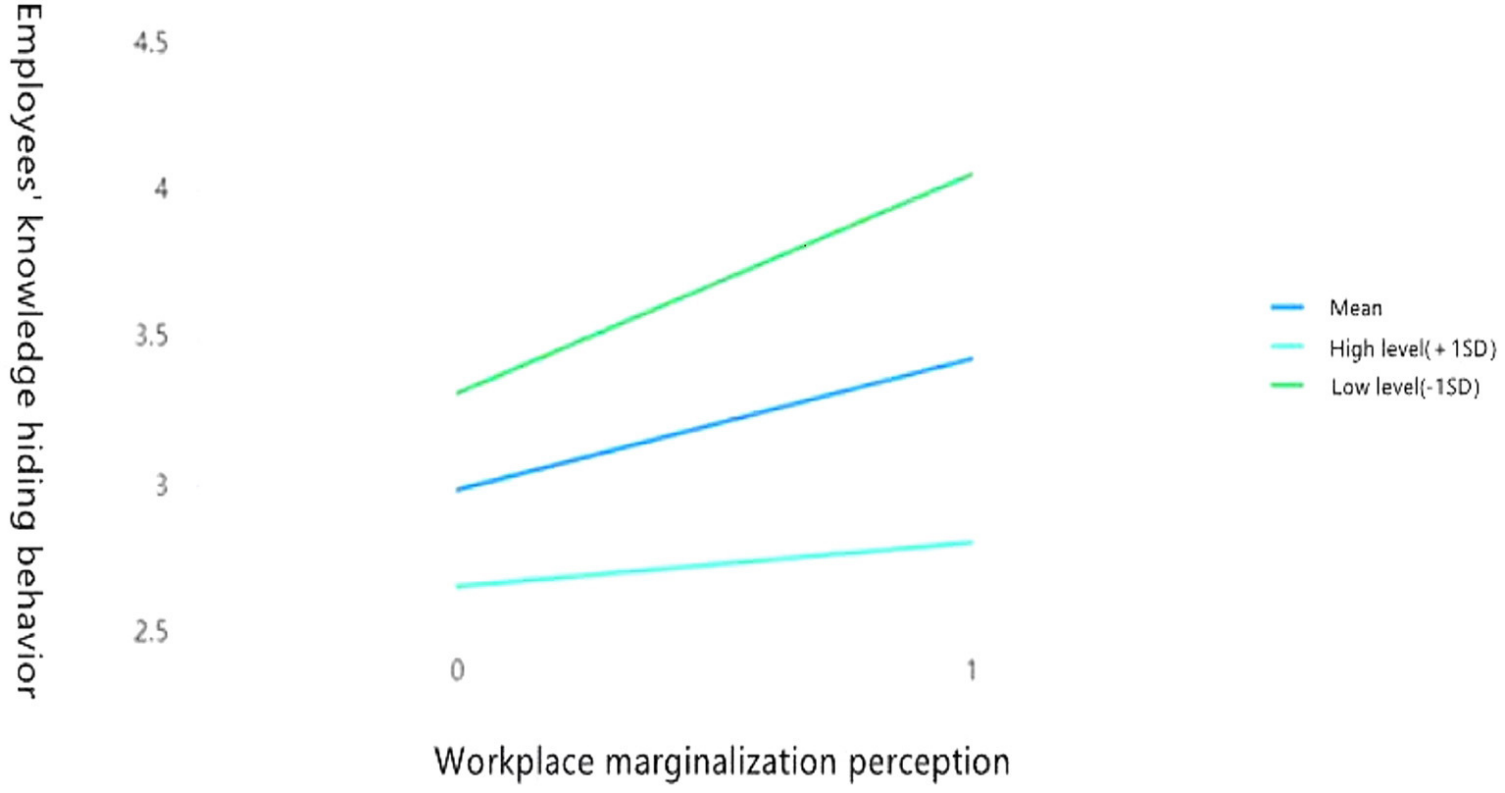
Emotional commitment to the organization plays a negative moderating role between workplace marginalization perception and employees' knowledge hiding behavior.

To analyze the negative moderating effects of high level of emotional commitment to the organization (M + 1 SD) and low level of emotional commitment to the organization (M – 1 SD), this study used the process plugin of SPSS v.22.0 to conduct a simple efficiency analysis, and drew a simple efficiency diagram of the moderating effect. The results are presented in Figures 2, 3.

As can be seen from Figure 2, the emotional commitment to the organization negatively moderates the influence of leaders' bias tendency on employees' knowledge hiding behavior [*β* = 0.130, *p* < 0.001, 95% confidence interval (0.069, 0.191), excluding 0]. When employees have the characteristics of high level of emotional commitment to the organization, the influence of the leaders' bias tendency on the employee's knowledge hiding behavior is *β* = −0.182 (*p* < 0.01), the 95% confidence interval is (−0.266, −0.097), excluding 0; when employees have the characteristics of low level of emotional commitment to the organization, the impact of leaders' bias tendency on employees' knowledge hiding behavior is *β* = 0.442 (*p* < 0.001), and the 95% confidence interval is (0.358, 0.524), excluding 0. This supports Hypothesis H5.

It can be seen from Figure 3 that emotional commitment to the organization negatively moderates the influence of workplace marginalization perception on employees' knowledge hiding behavior [*β* = 0.446, *p* < 0.001, 95% confidence interval (0.386, 0.507), excluding 0]. When employees have the characteristics of high level of emotional commitment to the organization, the impact of workplace marginalization perception on employees' knowledge hiding behavior is *β* = 0.148 (*p* < 0.001), and the 95% confidence interval is (0.058, 0.238), excluding 0; when employees have the characteristics of low level of emotional commitment to the organization, the impact of workplace marginalization perception on employees' knowledge hiding behavior is *β* = 0.744 (*p* < 0.001), and the 95% confidence interval is (0.662, 0.826), excluding 0. This supports Hypothesis H6.

## Discussion

### Summary

In the workplace, leaders' bias tendency can have a detrimental effect on employees. On the ground of multiple regression analysis and process analysis, this survey demonstrates that the leaders' bias tendency has an optimistic effect on employees' knowledge hiding behavior. Derived from the leader–member exchange theory and the resource conservation theory, we proposed a theoretical model of the behavior of leaders' bias tendency toward employees' knowledge hiding behavior. The findings support our hypothesis that the leaders' bias tendency is foremostly and helpfully related to employees' knowledge hiding behavior through workplace marginalization perception, and emotional commitment to the organization plays a negative moderating role. The results of the hypothesis test are shown in Table 6.

**Table 6:** The result of hypothesis test.

**Number**	**The content of hypothesis**	**Result**
H1	There is a positive correlation between the leaders' bias tendency and employees' knowledge hiding behavior.	Supported
H2	There is a positive correlation between the leaders' bias tendency and the workplace marginalization perception.	Supported
H3	There is a positive correlation between the workplace marginalization perception and the employees' knowledge hiding behavior.	Supported
H4	Workplace marginalization perception plays a mediating role between leaders' bias tendency and employees' knowledge hiding behaviors.	Supported
H5	Emotional commitment to the organization plays a negative moderating role between leaders' bias tendency and employees' knowledge hiding behaviors.	Supported
H6	Emotional commitment to the organization plays a negative moderating role between workplace marginalization perception and employees' knowledge hiding behavior.	Supported

### Theoretical contributions

The study has the following contributions: First, the study explores the influence of the leaders' bias tendency on employees' knowledge hiding behavior. Employees' knowledge hiding behavior has a compulsory hindering effect on knowledge sharing and organizational innovation within an organization. Existing research has explored the grounds of employees' knowledge hiding behavior, such as interpersonal doubt, psychological ownership of knowledge, employees' open personality, perception of organizational competitive climate, and sense of procedural fairness. However, the influence of the leadership type and leadership conduct on employees' knowledge hiding behavior is ignored. This study demonstrates that the leaders' bias tendency positively affects employees' knowledge hiding behavior. These studies strongly respond to the harm of negative leadership and the importance and value of avoiding these negative leadership behaviors.

Another contribution of the study is grounded on leader–member exchange theory and resource conservation theory, proving that workplace marginalization perception plays a significant part role between leaders' bias tendency and employees' knowledge hiding behavior, thus clarifying leaders' bias tendency to influence the mechanism of employees' knowledge hiding behavior.

Finally, the third contribution of this study is to combine the cultural and social factors to introduce emotional commitment to the organization as a moderator variable to adapt to the national conditions of China, where the study is located. The results of the study show that emotional commitment to the organization plays a negative moderating part between leaders' bias tendency and employees' knowledge hiding behavior; emotional commitment to the organization plays a negative moderating role between workplace marginalization perception and employees' knowledge hiding behavior, thereby expanding employees' knowledge hiding behavior. Therefore, this study explains the methods to alleviate employees' knowledge hiding behavior from a new perspective, and enriches the research on employees' knowledge hiding behavior.

### Limitations and recommendations for future research

Our study has the following constraints: (1) Limited by the resources available to the researchers, the effective sample size of this study is 500, which is relatively small. Fortunately, the entire samples in this study passed the dependability test, and the discriminant obvious between variables was good. In the future, we should consider adding more partners to help us increase the sample size and make the results of this study more reliable. (2) All information in this study come from employee self-evaluation, not employee–leader mutual evaluation. The reason for abandoning mutual evaluation is that the measured variable includes the leaders' bias tendency. Despite our commitment to the confidentiality of the research, many employees still reject leaders from directly participating in this research. To make up for the impact of the homologous method as much as possible, this study adopts the method of subsection survey, and the interval between the two surveys is 4 months. On the other hand, the samples in this study also passed Harmer single factor test, which suggests that there is no grievous common method bias in this study.

### Practical implications

In fact, this study shows that the leaders' bias tendency is an important reason for employees to produce knowledge hiding behavior. Leaders' bias tendency are expensive for organizations (Chen et al., 2014; Zhou et al., 2020), because employees' knowledge hiding behavior usually represents a decrease in employees' aspirations to share knowledge and an increase in the cost of knowledge dissemination within the organization (Wang et al., 2020). Therefore, the leaders of the organization should show solicitude for the impact of this phenomenon on the organization, give subordinates a sense of procedural fairness, and face the emergence of biased behavior and the emergence of differential order patterns. On the other hand, formulating more reasonable promotion and elimination channels can also effectively restrain the negative effect of the leaders' bias tendency (Du and Wang, 2022).

On the basis of leader–member exchange theory and resource conservation theory, this study also shows that workplace marginalization perception plays a mediating part between leaders' bias tendency and employees' knowledge hiding behaviors. Therefore, managers should consciously open the circulation channels of “insiders” and “outsiders” to reduce employees' workplace marginalization perception due to biased tendencies and differential patterns, so as to improve employees' opinions self-efficacy and encourage employees to share knowledge (Chen, 2020).

Finally, this study also shows that when employees' emotional commitment to the organization is high, the direct and profile effects of leaders' bias tendency and workplace marginalization perception on employees' knowledge hiding behavior are lower. Therefore, employees with high level of emotional commitment to the organization are less possible to exhibit knowledge hiding behaviors. Correspondingly, when employees' emotional commitment to the organization level is low, knowledge hiding behavior occurs more frequently. The emotional commitment to the organization of employees originates from the degree of recognition and maintenance of the organization in the employee's heart (Meyer, 2012). In China, traditional Confucian workplace values require employees to show loyalty and love to the organization (Wang and Zhang, 2012), which will enhance the level of employees' emotional commitment to the organization. Therefore, organizational leaders should consider introducing traditional Confucian workplace values into organizational culture to alleviate employees' knowledge hiding behavior.

## Data availability statement

The raw data supporting the conclusions of this article will be made available by the authors, without undue reservation.

## Ethics statement

The studies involving human participants were reviewed and approved by the Ethics Committee of Qinghai University. The patients/participants provided their written informed consent to participate in this study. Written informed consent was obtained from the individual(s) for the publication of any potentially identifiable images or data included in this article.

## Author contributions

All authors listed have made a substantial, direct, and intellectual contribution to the work and approved it for publication.

## Conflict of interest

The authors declare that the research was conducted in the absence of any commercial or financial relationships that could be construed as a potential conflict of interest.

## Publisher's note

All claims expressed in this article are solely those of the authors and do not necessarily represent those of their affiliated organizations, or those of the publisher, the editors and the reviewers. Any product that may be evaluated in this article, or claim that may be made by its manufacturer, is not guaranteed or endorsed by the publisher.
